# The roles of osteoprotegerin in cancer, far beyond a bone player

**DOI:** 10.1038/s41420-022-01042-0

**Published:** 2022-05-06

**Authors:** Yufei Wang, Yiyang Liu, Zhao Huang, Xiaoping Chen, Bixiang Zhang

**Affiliations:** 1 Hepatic Surgery Center, Tongji Hospital, Tongji Medical College, Huazhong University of Science and Technology, Wuhan, China; 2 Clinical Medical Research Center of Hepatic Surgery at Hubei Province, Wuhan, China; 3 Hubei Key Laboratory of Hepato-Pancreatic-Biliary Diseases, Tongji Hospital, Tongji Medical College, Huazhong University of Science and Technology, Wuhan, China; 4 Key Laboratory of Organ Transplantation, Ministry of Education; Key Laboratory of Organ Transplantation, National Health Commission; Key Laboratory of Organ Transplantation, Chinese Academy of Medical Sciences, Wuhan, China

**Keywords:** Tumour biomarkers, Cancer

## Abstract

Osteoprotegerin (OPG), also known as tumor necrosis factor receptor superfamily member 11B (TNFRSF11B), is a member of the tumor necrosis factor (TNF) receptor superfamily. Characterized by its ability to bind to receptor activator of nuclear factor kappa B ligand (RANKL), OPG is critically involved in bone remodeling. Emerging evidence implies that OPG is far beyond a bone-specific modulator, and is involved in multiple physiological and pathological processes, such as immunoregulation, vascular function, and fibrosis. Notably, numerous preclinical and clinical studies have been conducted to assess the participation of OPG in tumorigenesis and cancer development. Mechanistic studies have demonstrated that OPG is involved in multiple hallmarks of cancer, including tumor survival, epithelial to mesenchymal transition (EMT), neo-angiogenesis, invasion, and metastasis. In this review, we systematically summarize the basis and advances of OPG from its molecular structure to translational applications. In addition to its role in bone homeostasis, the physiological and pathological impacts of OPG on human health and its function in cancer progression are reviewed, providing a comprehensive understanding of OPG. We aim to draw more attention to OPG in the field of cancer, and to propose it as a promising diagnostic or prognostic biomarker as well as potential therapeutic target for cancer.

## Facts

- OPG is well-recognized for its participation in bone homeostasis and the skeletal metastasis of cancer.
- Emerging evidence has revealed versatile roles of OPG in human physiological and pathological processes, such as vascular formation, immune modulation and fibrosis.
- Except for the RANKL and TRAIL, the regulation of OPG functional activity and expression levels are modulated by various other ligands and signaling pathways.
- OPG is widely expressed in numerous types of cancer cells. Advances in the field of cancer have unveiled the diagnostic, prognostic and therapeutic value of OPG in various cancers, especially bone metastasis.
- The roles of OPG in cancer are far from clear, and more research is needed to completely elucidate the involvement of OPG in cancers.

## Open Questions

- The published results regarding the impact of OPG on some types of cancer are inconsistent and even contradictory. How can these discrepancies be explained?
- RANKL and OPG are considered antagonistic effectors in most cases. However, independent researches studies have shown that they may fulfil concurrent functions in cancers. Do these two proteins have conflicting therapeutic applications in cancers? How can we better understand the relationship between these two cancer players?
- The levels of OPG in different forms and compartments (mRNA, intracellular OPG protein and serum OPG protein) are often discordant. What is the molecular mechanism underlying this discrepancy? Which index is most important for cancer diagnosis or prognosis?
- As a secreted protein, OPG is competent for acting as a signaling molecule between different cells. Is it more important to identify the main source of secreted OPG in a particular micro-environment than to focus on the expression pattern of OPG in cancer cells?
- How can the antitumor effect of OPG be selectively improved to broaden its therapeutic prospects?

## Introduction

**Fig. 1:**
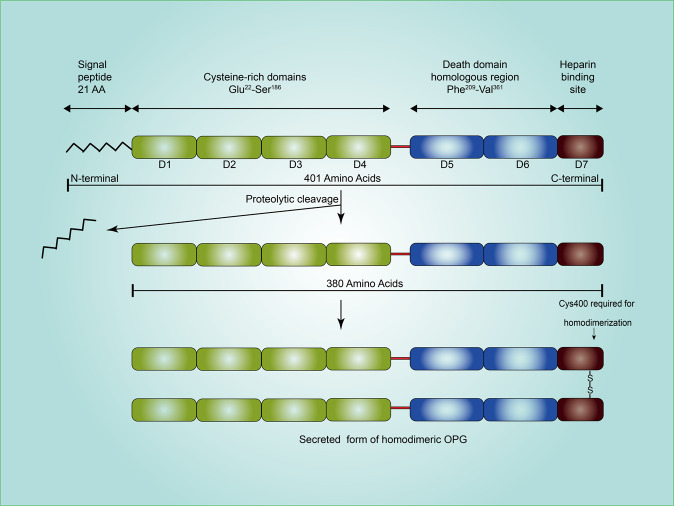
Molecular structure and maturation process of secreted OPG. The signaling peptide is removed from full length OPG molecule before being secreted. The secreted OPG is a homodimer linked by disulfide bonds. AA, amino acid, D (1–7) domain (1–7).

As a member of the TNF receptor superfamily, OPG is well recognized for its protective effect against excessive bone resorption [1]. Overall, OPG protein contains a signal peptide and seven functional domains. After proteolytic cleavage of the signal peptide and homodimerization, the mature form of OPG is secreted from the cytoplasm to the extracellular compartment [2] (Fig. 1).

**Fig. 2:**
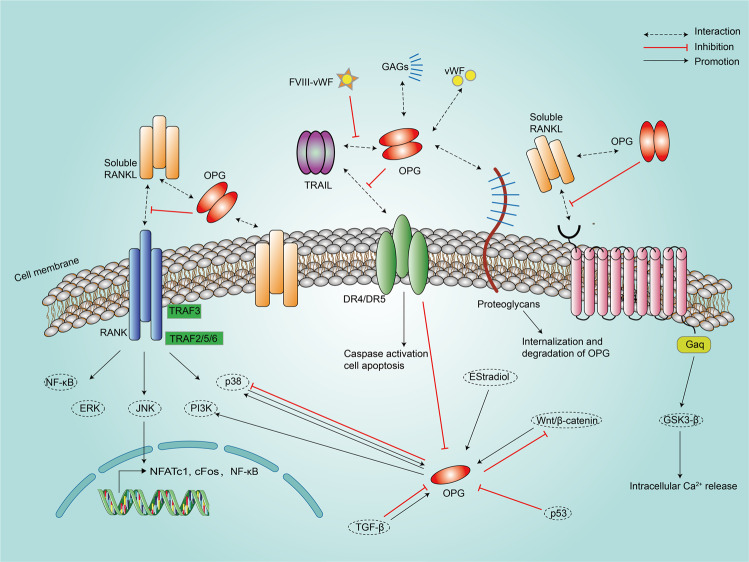
OPG-related signaling pathways. OPG is secreted in a homodimeric form. By binding with RANKL and TRAIL, OPG exerts its inhibitory effects on their downstream signaling pathways. Besides, OPG itself is regulated by many signaling pathway. Imbalanced signaling cross-talks between PI3K/AKT, p38/MAPK, wnt/β-catenin, TGF-β, mTOR and OPG contribute to the pathological cell behaviors. Other ligands, such as E2, vWF, FVIII-vWF and glycosaminoglycans (GAGs), bind with secreted OPG to impede its function. DR death receptor.

By binding to RANKL, OPG suppresses the maturation and activation of pre-osteoclasts *via* antagonizing RANK/RANKL signaling (Fig. 2), thus inhibiting osteoclast function and promoting bone formation [3]. Therefore, attenuated OPG expression or an impaired RANKL/OPG ratio results in osteoporosis [4], rheumatoid arthritis [5], or periodontitis [6]. Another well-recognized endogenous ligand of OPG is TNF-related apoptosis-inducing ligand (TRAIL). OPG hinders the interaction between TRAIL and its death receptors (DRs), and thus blocks TRAIL-induced apoptosis [7] (Fig. 2). The crucial roles of RANKL and TRAIL signaling in the development of the immune system and various stages of tumor progression potentiate OPG as a key player in cancer formation and progression. In fact, as a secreted protein, the clinical significance of OPG has been studied in several tumors including breast cancer, prostate cancer (PCa), multiple myeloma (MM), and hepatocellular carcinoma (HCC). In these studies, OPG exhibits its capacity as a predictive index of bone metastasis, a diagnostic or prognostic marker of malignancies. Moreover, the therapeutic effect of OPG was investigated in established mouse models of MM [8, 9] and in tumor bone metastasis [10] by using exogenous bioactive OPG.

## Molecular structure and expression of OPG

Independently identified by Simonet et al. [1] and Tsuda et al. [11] in 1997, OPG was previously named osteoclastogenesis inhibitory factor (OCIF), TNF receptor-related molecule-1 (TR-1) or follicular dendritic cell receptor-1 (FDCR-1). It was eventually named osteoprotegerin (OPG) in 2000 [12], due to its major function in bone homeostasis. OPG is encoded at chromosome 8q23-24, and is a secreted protein without a transmembrane region [3]. Biochemically, full length OPG protein, with a monomeric weight of 55–62 kDa, is 401 amino acids long and contains a signal peptide of 21 amino acids, which is then proteolytically cleaved to yield a 380 amino acid form (Fig. 1). There are four cysteine-rich N-terminal domains (domains 1–4), two death domain homologous regions (domains 5 and 6), and a C-terminal heparin-binding domain (domain 7) [2] (Fig. 1). OPG is secreted as a homodimer linked by disulfide bonds within 4 or 5 potential glycosylation sites, resulting in a mature secreted form of 110–120 kDa. In this process, the cysteine at position 400 is essential for homodimerization. The OPG dimer has the highest heparin-binding ability and the highest calcium-lowering ability [3].

Different cells express and secrete OPG in bone marrow, including osteoblasts, B cells, megakaryocytes, platelets, vascular endothelial cells, and vascular smooth muscle cells [1]. In addition to skeletal expression, OPG expression is also detected in many tissues including the heart, kidney, liver and spleen [13].

### Physiological function of OPG

#### Bone remodeling

The delicate balance between osteolytic and osteoblastic activity in bone depends mainly on two major cell types: mesenchymal stem cells (MSC) derived osteoblasts and myeloid lineage-derived osteoclasts [2, 14]. The RANKL/RANK/OPG axis is the utmost determinant of bone remodeling. Total bone mineral density is decreased in OPG^−/−^ mice, resulting in severe trabecular and cortical porosity, thin parietal bone, and a high incidence of fractures [15]. The bone protective effect of OPG is also supported by a report of a 100,000-bp homozygous deletion of OPG in two young patients with Paget’s disease, an autosomal recessive disorder characterized by increased bone remodeling, osteopenia and frequent fractures [16]. The imbalance of OPG/RANKL leads to a series of bone diseases including osteoporosis, rheumatoid arthritis, and periodontitis, manifesting as excessive activation of osteoclast activity and an increased RANKL/OPG ratio [6, 17, 18]. Numerous literatures have detailed the functions of OPG in skeletal disease, but this topic is not the main focus of this review and can be referred to Kearns et al. [19].

#### Vascular biology

The function of OPG in vascular biology was first revealed by the observation that OPG-deficient mice suffered marked calcification of the aorta and renal arteries [15]. Di et al. reported that OPG regulated insulin-like growth factor 1 receptor (IGF1R) expression and activity could modulate vascular smooth muscle cell calcification in vitro [20]. Deletion of OPG in apolipoprotein E knockout mice accelerated calcified atherosclerosis, suggesting that OPG can prevent such process of atherosclerosis [16]. Gordin et al. reported that serum OPG was an independent indicator of cardiovascular complications in adults with type 1 diabetes; OPG might play a role in extraosseous calcification, resulting in atherosclerosis and subsequent vascular dysfunction [21]. Moreover, elevated serum OPG levels were detected in patients with carotid calcification [22]. Furthermore, serum OPG levels demonstrated a prognostic value independent of other known predictors of mortality and cardiovascular events in patients with heart failure after acute myocardial infarction [23].

#### Immunity modulator

OPG-dependent self-regulation of microfold cell differentiation was discovered by Kimura et al. [24], who demonstrated that OPG expressed by microfold cells suppressed the differentiation of adjacent follicle-associated epithelial cells into microfold cells, and thus created a weakened immune microenvironment [24]. The accumulation of type 1 transitional B cells and isotype class switch defects were observed in OPG^−/−^ mice, suggesting the role of OPG in regulating B cell maturation and development [25]. In vitro experiments revealed that, OPG activated Akt/PI3K signaling pathway in endothelial cells (ECs), subsequently leading to the recruitment and adhesion of monocytes [26]. OPG mediated negative regulation of the transcription factor Spi-B during the development of medullary thymic epithelial cells weakened the proliferative ability of regulatory T cells and inhibited tumor development in mice [27], suggesting a regulatory role of OPG in antitumor immunity.

#### Fibrosis

Recently, OPG has been found to be involved in fibrosis, which is characterized by the excessive formation and accumulation of extracellular matrix proteins generated by myofibroblasts [28]. The administration of exogenous OPG to chondrosarcoma cells lowered the expression of both mRNA and protein of matrix metalloprotease-13 [29], which is considered an important anti-fibrotic factor. Adhyatmika et al. reported that OPG produced by liver tissue promoted fibrogenesis in a transforming growth factor-β (TGF-β) dependent manner, and this was restrained by treatment with the TGF-β receptor kinase inhibitor galunisertib (LY2157299) [30]. To develop noninvasive approaches to assess liver fibrosis in patients with chronic liver diseases, Bosselut et al. constructed a score-based blood test for liver fibrosis involving OPG, which showed an improved diagnostic accuracy compared to existing scoring systems [31]. Compared with healthy livers, fibrotic livers in humans and mice had increased OPG levels, resulting in an increased expression of genes associated with fibrogenesis through TGF-β1 [32].

### Regulation of OPG expression

As one of the most important ligands of OPG, RANKL produced by osteoblasts binds to RANK on the surface of osteoclast precursors and recruits the cohesive protein TRAF6, resulting in the activation of NF-κB and translocation to the nucleus. This subsequently leads to the increased expression of c-Fos, which interacts with nuclear factor of activated T cells c1 (NFATc1) to trigger the transcription of osteoclast genes [16]. The downstream signaling pathway initiated by RANKL activation also includes PI3K, ERK, JNK, and p38/MAPK [33] (Fig. 2). Besides, a membrane receptor called leucine-rich repeat-containing G-protein-coupled receptor 4 (LRG4) of RANKL was recently identified. RANKL binds to the extracellular domain of LGR4 and negatively regulates osteoclastogenesis by activating Gαq/GS3K-β signaling and inhibiting the NFATc1 pathway [2]. OPG restrains this process by binding to extracellular RANKL (Fig. 2). Meanwhile, screening of OPG protein with TNF-related ligands resulted in the discovery that OPG binds to TRAIL, thus preventing its interaction with death receptors and blocking TRAIL-induced apoptosis in a human T lymphocyte cell line named Jurkat [7]. Reciprocally, OPG mRNA expression was significantly inhibited by TRAIL stimulation in normal human bone marrow-derived stromal cells [34] (Fig. 2).

Numerous signaling molecules or pathways have been demonstrated to regulate the expression of OPG (Fig. 2). Boyce et al. reported that β-catenin together with T-cell factor proteins regulated the expression of OPG in mouse osteoblasts [35]. TGF-β upregulated OPG and downregulated RANKL in osteoblasts through the canonical β-catenin dependent Wnt signaling pathway [28, 36]. However, the exogenous administration of TGF-β1 to oral squamous cell carcinoma cells upregulated RANKL and downregulated OPG [37]. Additionally, the activation of p38MAPK signaling increased the expression of OPG in HCC and mouse mesenchymal stem cells (MSCs) [38, 39]. However, Hao et al. demonstrated that the OPG expression was negatively regulated by p38MAPK in pre-osteoblast cells [40]. The female sex hormone estradiol was found to upregulate OPG and thus interfere with RANKL/RANK signal activation, which explains the tendency of elderly women to suffer from osteoporosis due to the decreased production of estradiol [41]. The deletion of Notch1 in osteoblast lineage cells promoted osteoclastogenesis by decreasing the OPG/RANKL expression ratio [42]. Qiang et al. reported that Wnt3a-regulated OPG and RANKL expression was disrupted by MM cell-derived DKK1, a soluble inhibitor of canonical Wnt signaling in osteoblasts [43].

In addition to RANKL and TRAIL, OPG was also found to bind to von Willebrand factor (vWF), and was subsequently stored within Weibel-Palade (WP) bodies, which fused with the outer membrane of endothelial cells and released their contents into the plasma when activated [44]. Likewise, the FVIII/vWF complex is capable of binding to OPG, which blocks the interaction of OPG with TRAIL, indicating the potential of the FVIII/vWF complex in tumorigenesis [26]. Heparan sulfate proteoglycans on the surface of cultured myeloma cell lines were found to bind to OPG, subsequently leading to its internalization and degradation [45].

### OPG mediated hallmarks of cancer

OPG was found to prevent cell apoptosis and promote tumor survival. Lane et al. reported that OPG inhibited the TRAIL-mediated apoptosis of ovarian cancer cells in an α_v_β_3_ and α_v_β_5_ integrin-dependent manner [46]. In inflammatory breast cancer, OPG interacted with glucose-regulated protein/binding immunoglobulin protein (GRP78/BiP), the endoplasmic reticulum (ER) chaperone and master regulator of the unfolded protein response, to promote cell apoptosis and prolong the survival of inflammatory breast cancer cells [47]. By forming complexes with the lipid raft core protein Cav-1 and EGFR, RANK promoted gastric cancer cell migration, and this effect was mitigated by OPG [48]. Recombinant OPG (rOPG) restrained breast cancer stemness by suppressing the β-catenin pathway, thus inhibiting tumor growth, EMT, and metastasis in orthotopic breast cancer xenografts [49].

The role of OPG has also been elaborated in angiogenesis, a process essential for the maintenance, development, and progression of malignancies. Cross et al. reported that the expression of OPG in the endothelium of malignant colorectal, breast, and metastatic cancer tumors was higher than that in benign tumors or in normal tissues. Exogenous stimulation with OPG facilitated the formation of cord-like structures by endothelial cells in vitro [50]. In addition, α_v_β_3_ was found indispensable for the resistance to apoptosis by administration of rOPG in human microvascular endothelial cells [51]. Benslimane et al. reported that OPG combined with fibroblast growth factor (FGF-2) promoted neovascularization in vivo, accompanied by the activation of proangiogenic pathways involving MAPK signaling pathway, as well as Akt or mTOR cascades in endothelial colony forming cells (ECFCs) [52]. Moreover, OPG-pretreated ECFCs secreted stromal cell-derived factor-1 (SDF-1), which promoted neovascularization in ischemic tissue [53].

### Role of OPG in cancers

#### OPG in cancer bone metastasis

Bone is one of the most common sites of cancer metastasis, especially for prostate, breast, and lung cancer [54]. Given the crucial role of OPG in bone homeostasis, the involvement of OPG in cancer bone metastasis has been widely investigated. Among the bone markers tALP, NTX, BSP, P1NP, bALP, TRAP, and OPG, this last marker displayed the highest sensitivity and accuracy for identifying bone metastases of PCa [55]. In the reseach of benign prostatic hyperplasia, primary and metastatic PCa disease, elevated OPG mRNA expression was found in metastatic tumor tissues compared to local disease. A higher RANKL/OPG ratio was observed in tumors with bone metastases than in normal prostate tissues [56]. Using conditionally replicating adenoviruses (CRAds), Jung et al. demonstrated that overexpressing OPG suppressed osteoclastogenesis in vitro and inhibited the progression of advanced PCa bone metastases [57]. Corey et al. reported that increased expression of OPG in C4-2 PCa cells did not inhibit its proliferation directly, but suppressed the growth of skeletal metastatic PCa tumors [58]. By testing serum OPG and RANKL levels, Elfa et al. concluded that the sensitivity and specificity of serum OPG in detecting bone metastasis in breast cancer patients were 59% and 92%, respectively, while the corresponding values for the RANKL/OPG ratio were 73% and 72%, respectively [59]. In liver cancer, Huang et al. reported that the long noncoding RNA H19 promoted HCC bone metastasis by attenuating OPG expression to create a pro-metastatic bone microenvironment [39].

With the ability to inhibit osteoclast activity and prevent the vicious cycle of osteoclast-tumor cell interactions, bisphosphonates are commonly administered to clinical patients with bone metastases. Rachner et al. found that the TRAIL/OPG ratio was increased by zoledronic acid (ZA) in TRAIL-sensitive MDA-MB-231 cells, suggesting that the TRAIL/OPG cytokine system is among the bisphosphonate-responsive targets in breast cancer in vitro [60]. After 12 months of treatment with ZA, a decrease in the RANKL/OPG ratio was observed, indicating that ZA decreased osteoclast activity by modulating RANKL and OPG expression [61].

#### OPG in breast cancer

A nested case control study in the European Prospective Investigation into Cancer and Nutrition (EPIC) cohort revealed that higher serum OPG levels might be a novel risk factor for ER-negative breast cancer [62]. In addition, in the EPIC cohort, Sarink et al. found that high concentrations of serum OPG were associated with elevated breast cancer specific and overall mortality, especially in those with ER-positive breast cancer [63]. In the Tromsø study, serum OPG was correlated with a reduced risk of breast cancer in women <60 years after adjustment. However, a worse prognosis was associated with patients with OPG levels in the upper quartile [64]. RANKL and OPG levels were reported to be upregulated in patients with breast cancer, and OPG was associated with the burden of bone metastases according to Mountzios et al. [65]. While BRAC1/2 mutation is a risk factor for breast cancer, lower serum OPG levels were correlated with the germline mutations of BRAC1/2 known to facilitate breast cancer risk [66]. Musculoskeletal toxicity is the major adverse effect of aromatase inhibitor therapy, which significantly affects the quality of life of patients. Lintermans et al. found that the SNP rs2073618 in OPG was associated with an elevated risk of musculoskeletal symptoms and pain after aromatase inhibitor therapy [67].

#### OPG in prostate cancer

Based on a strong negative correlation between endogenous OPG levels and TRAIL-induced apoptosis in vitro, Holen et al. concluded that OPG produced by PCa might be a considerable survival factor in hormone-resistant PCa cells [68]. In addition to predicting bone metastasis, OPG was found by multivariate regression analysis to be an independent prognostic factor for PCa related death [55]. Conditioned medium from PCa cells stimulated osteoclast formation, and this effect could be weakened by pre-exposure of osteoblasts to NF-κB inhibitor parthenolide (PTN), which reduced RANKL/OPG ratio [69]. By analyzing the correlation of p53 and the OPG/RANKL axis in PCa, Velletri et al. demonstrated that the loss of p53 was associated with high OPG levels both in vivo and in vitro, and that this predicted poor prognosis [70].

#### OPG in multiple myeloma

As one of the major features of MM, osteolytic lesions are caused by the excessive activation of osteoclasts and inhibition of osteoblasts [71]. Therefore, the imbalance in OPG/RANKL signaling has attracted much attention. Goranova-Marinova et al. found that serum OPG, RANKL, and RANKL/OPG ratios were significantly higher in MM patients than in healthy controls [72]. Inactivation of osteoclasts and trabecular bone loss in the vertebrae and tibiae was observed in mice following systemic administration of OPG expressing MSCs, suggesting the protective effect of OPG in MM [73].

OPG/RANKL is also used as a marker of bone remodeling. After the administration of two doses of lenalidomide, serum RANKL level, as well as the RANKL/OPG ratio, was significantly reduced, whereas serum OPG was elevated [71]. In a study of 104 patients who received at least 1 cycle of bortezomib, serum RANKL declined significantly, but serum OPG levels remained unchanged, causing a drop in the RANKL/OPG ratio [74]. The therapeutic effect of ZA was evaluated by measuring bone turnover markers including OPG. Interestingly, OPG levels in gingival crevicular fluid (GCF) were higher after 3 months of ZA therapy in patients with 5 or more bone metastases [75]. After analyzing the genotypes of 3774 MM patients of European ancestry, rs4407910 at 8q24.12 (odds ratio = 1.38, *P* = 4.09 × 10^−9^) and rs74676832 at 19q13.43 (odds ratio = 1.97, *P* = 9.33 × 10^−7^) showed the strongest association with MM bone disease (MBD). These data further support and highlight the role of OPG in the development of MM [76].

#### OPG in hepatocellular carcinoma

By analyzing 40 pairs of tumor tissues and corresponding adjacent normal tissues utilizing real-time PCR, Jiang et al. found that RANK and OPG levels were elevated in tumor tissue [77]. The prognostic value of serum OPG in HCC was reported by Zhang et al., who revealed that high serum OPG levels were correlated with worse prognosis and OPG was an independent risk factor for liver cancer [78]. A prediction model for early stage hepatitis B-related liver cancer containing five proteins including OPG was established by Cheng et al. [79], demonstrating the potential diagnostic value of OPG.

Gao et al. observed lower mRNA expression of OPG in highly metastatic HCC cell lines (MHCC97H, HCCLM3, and Sk-Hep1) compared to lines with low metastatic potential (HuH7 and HepG2) [80]. Long non-coding RNA H19 was found to promote HCC bone metastasis by inhibiting OPG expression [39]. These results implied that OPG may be an anti-metastatic factor in HCC. Interestingly, MHCC97-L cells expressed higher levels of OPG protein under hypoxic conditions, despite lower levels of OPG mRNA [80]. This inconsistency between the protein and mRNA expression levels may be attributed to the special properties of OPG as a secreted protein.

#### Clinical relevance of OPG in other cancers

The mRNA and protein expression of OPG was found to be upregulated in pancreatic cancer compared to normal pancreatic tissues, and both univariate and multivariate analyses demonstrated that the elevated levels of OPG were adversely correlated with the overall survival of cancer patients [81]. Aversa et al. showed that OPG was one of 12 biomarkers that were positively correlated with esophageal squamous cell carcinoma risk [82]. For oral squamous cell carcinoma, the elevated OPG expression was shown to be associated with bone invasion and shorter long-term cancer-specific survival [83] (Table 1).

**Table 1:** A glance of clinical significance of OPG in cancers.

Cancer type	Analysis	Diagnostic value	Prognostic value
ESCC [82]	PEA	Increased ESCC risk with higher OPG level	Not available
HCC [78]	ELISA	Not available	Higher serum OPG associated with worse overall-survival
m-ccRCC [95]	RT-PCR	Not available	Increased RANK/OPG ratio associated with BM and worse prognosis
OSCC [83]	IHC	Not available	Elevated OPG expression associated with bone invasion, poor pathological tumor regression to neoadjuvant CRT, and worse long-term cancer-specific survival
BC and GC [64]	ELISA	Reduced of breast cancer risk and increase GC risk with higher serum OPG	Upper tertile of OPG with higher risk of cancer-related mortality particularly for GC
BC [59]	ELISA	Bone metastasis with reduce OPG level	Not available
BC [62]	ELISA	Increased risk of ER^-^ BC with higher serum OPG	Not available
BC [63]	ELISA	Not available	Higher OPG associated with increased mortality after a BC diagnosis, particularly in ER^+^ patients
PCa [55]	ELISA	Bone metastases with higher OPG level	Higher serum OPG associated with worse prognosis
PCa [56]	RT-PCR	Elevated OPG mRNA expression in metastatic tumor compared to BPH	Not available
MM [72]	ELISA	Higher OPG and RANKL/OPG ratios in MM patients	Not available
PaC [81]	IHC	Upregulated OPG in PaC tissues	Upregulated OPG with poor overall survival

*BC* breast cancer, *BPH* benign prostatic hyperplasia, *ELISA* enzyme linked immunosorbent assay, *ER* estrogen receptor, *ESCC* esophageal squamous cell carcinoma, *GC* gastrointestinal cancer, *HCC* hepatocellular carcinoma, *IHC* immunohistochemistry, *m-ccRCC* metastatic clear-cell renal carcinoma, *MM* multiple myeloma, *OSCC* oral squamous cell carcinoma, *PCa* prostate cancer, *PaC* pancreatic cancer, *PEA* proximity extension assay, *RT-PCR* real-time polymerase chain reaction.

### Translational endeavors of OPG for cancer treatment

As early as 2001, the therapeutic potential of OPG was reported. Vanderkerken et al. found that the tumor burden of the recombinant OPG treatment group was reduced and the tumor onset time was delayed in MM murine model [8]. A recombinant OPG construct was also reported to inhibit bone resorption at the molecular level, which manifested as a decrease in the bone resorption marker urinary N-telopeptide of collagen (NTX) in patients with MM [9]. A mouse model of osteosarcoma expressing truncated OPG showed lower tumor incidence and longer survival [84]. However, OPG failed to inhibit pulmonary metastasis [84], illustrating the relationship of OPG with the bone microenvironment on OPG. What’s more, OPG-Fc treatment of mice with established bone metastases positively impacted prognosis [85]. After the administration of OPG-Fc to 12-week-old BALB/c mice, ovariectomy-induced osteoclast lesions and growth of disseminated tumor cells were prevented. This finding might suggest that bone metastases can be prevented by early administration of anti-resorptive therapy, including OPG, in a low-estrogen environment [86]. The capability of rAAV-OPG therapy to inhibit tumor growth and protect bone integrity was elucidated in a mouse model of bone metastasis [87]. In addition, OPG-Fc inhibited the occurrence and progression of bone metastases in mice bearing skeletal non-small cell lung cancer tumors [10]. Treatment with rOPG-Fc prevented bone damage by patient-derived B cell acute lymphoblastic leukemia (B-ALL) xenografts, even under conditions of a heavy tumor load [88].

In addition to binding to RANKL to inhibit bone resorption, OPG also combines with TRAIL to exert an anti-apoptotic effect, thus limiting the clinical applications of OPG in cancer patients. Special genetically engineered MSCs overexpressing OPG that selectively bind to RANKL, but not to TRAIL were designed by Qiang et al. As expected, the osteoclast activity induced by tumor cells expressing mutant OPG was significantly suppressed, suggesting the potential therapeutic value of this technology [89]. This provided clues for us to develop OPG-related therapeutics with fewer side effects and greater efficacy.

Due to the antagonistic relationship between OPG and RANKL, inhibiting RANKL may somewhat mimic the effect of OPG. The clinical development of OPG-Fc could be replaced by a human monoclonal antibody called denosumab (AMG 162), which specifically inhibits RANKL [90]. Based on data from considerable preclinical studies, many clinical trials have been conducted for denosumab and certain phase III clinical trials achieved exhilarant outcomes in patients with early stage breast cancer or newly diagnosed MM [90–94].

## Conclusions

**Fig. 3:**
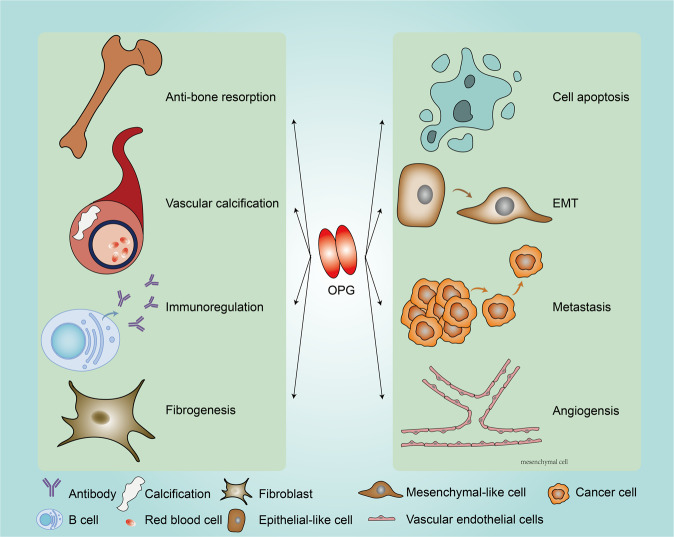
Summary of the role of OPG in benign and malignant diseases. OPG is involved in various physiological and pathological steps for disease progression, including bone remodeling, vascular calcification, angiogenesis, immunoregulation, fibrosis, cell survival and apoptosis, EMT, and cancer metastasis.

The roles and molecular mechanisms of OPG in bone biology and pathology have been intensively investigated in the past decades. Beyond its involvement in bone diseases, comprehensive understanding of OPG in other benign diseases and in cancer progression has been achieved. OPG is a multifaceted player that can regulate vascular biology, fibrosis, immunity, EMT, and the apoptosis of cancer cells (Fig. 3). As a secreted protein, OPG exerts bioactive functions by modulating various signal transduction pathways in cells, as well as remodeling the local microenvironment. At the same time, the presence of secreted OPG in human blood and the unique expression pattern provide a potential non-invasive approach to the diagnosis and prognosis of cancer patients. The function of OPG is contextual, given it influences numerous signaling, which exerts contradictory functions for cancer progression. Thus, it is essential to modify the endogenous OPG. Engineered OPG should display selective targeting effects to avoid synchronous side effects.
